# “Living with” *CACNA1A*-related hemiplegic migraine, a disease concept model

**DOI:** 10.3389/fneur.2024.1460187

**Published:** 2024-11-01

**Authors:** Donna Schaare, Kendra Allison, Kara Skorge, Pangkong Fox, Laina Lusk, Sara M. Sarasua, Ingo Helbig, Luigi Boccuto

**Affiliations:** ^1^Ph.D. Program in Healthcare Genetics and Genomics, School of Nursing, College of Behavioral, Social and Health Sciences, Clemson University, Clemson, SC, United States; ^2^School of Nursing, College of Behavioral, Social and Health Sciences, Clemson University, Clemson, SC, United States; ^3^Pfizer Medical Affairs, New York, NY, United States; ^4^Perlman School of Medicine, University of Pennsylvania, Philadelphia, PA, United States; ^5^CACNA1A Foundation, Inc., Norwalk, CT, United States; ^6^Division of Neurology, Children’s Hospital of Philadelphia, Philadelphia, PA, United States; ^7^The Epilepsy NeuroGenetics Initiative (ENGIN), Children’s Hospital of Philadelphia, Philadelphia, PA, United States; ^8^Department of Neurology, Perelman School of Medicine, University of Pennsylvania, Philadelphia, PA, United States

**Keywords:** *CACNA1A*, hemiplegic migraine, disease concept model, impacts, symptoms, caregivers

## Abstract

**Introduction:**

*CACNA1A*-related Hemiplegic Migraine (HM) is a rare neurological disorder distinguished by paroxysmal episodes of hemiparesis/hemiplegia with and without headache. Clinical features have been widely characterized, yet the impacts of the paroxysmal events on the patient and caregiver have not been thoroughly explored. Disease concept models are formal frameworks used to describe the lived experiences of patients and their families, offering a source for surrogate endpoints for clinical trials.

**Methods:**

We completed 13 semi-structured interviews with caregivers of 12 individuals diagnosed with *CACNA1A*-related HM. We methodically coded themes, grouping concepts into three domains. We measured the occurrence of concepts throughout all interviews and subgroups stratified by age categories.

**Results:**

Over 11 h of interviews yielded 2,018 references to 27 distinct concepts. Established symptoms such as seizures (87 references; including status epilepticus 27 references), hemiparesis/hemiplegia (24 references), and unconsciousness (17 references) were referenced, as well as previously underreported symptoms such as apneic episodes (32 references), lost ability to eat (13 references), and vascular access challenges (10 references). The symptom impacts were largely medical (294 references), followed by health (101 references), emotional (36 references), daily living (28 references), and social (26 references). Caregiver impacts were the most referenced domain (995 references), with the pivotal effects seen in caregiver requirements (355 references), emotional (245 references), HM treatments (179 references), daily living (148 references), and health support (135 references).

**Discussion:**

*CACNA1A*-related HM is a complex disorder defined by serious paroxysmal events that affects a broad range of social and clinical domains. We systematically classified symptoms and impacts from HM episodes, creating a disease concept model to help develop surrogate endpoints for future clinical trials, and identified two opportunities to improve patient management, including a written emergency protocol and a transition plan for adolescents approaching adulthood.

## 1 Introduction

*CACNA1A*-related disorders are rare neurological manifestations caused by pathogenic variants in the *CACNA1A* gene that include a broad spectrum of phenotypes, including epilepsy, developmental and epileptic encephalopathies, intellectual disability, autism spectrum disorder, ataxia, and hemiplegic migraine (1–3). HM events are paroxysmal and are often life-threatening, including hemiplegia, status epilepticus, coma, cerebral edema, encephalopathy, fever, and apnea (4–15). These episodes require immediate identification and action to avoid unwanted adverse patient outcomes. Currently, there are no Food and Drug Administration (FDA)-approved therapeutics specifically designed for treating and preventing these life-altering events. Instead, clinicians must rely upon anecdotal case reports or very small cohorts to inform management protocols (16). Current treatments focus on administering multiple doses of proposed rescue medications in the hopes of early abortion of HM episodes. This requires caregivers to identify the subtle signs during the initiation of events, which is difficult for individuals with communication challenges.

Much of the HM phenotype has been described, focused primarily on the milder phenotype seen in familial cases (FHM1) that can be managed using prophylactic treatments such as calcium channel blockers, acetazolamide, and other antiseizure medications especially in patients with a predominant migraine pattern and epilepsy (17, 18). These cases tend to be neurotypical at baseline and can present with HM events as early as childhood or much later into adulthood (17). The more severe phenotype, often apparent in sporadic cases (SHM1), coincide with neurodevelopmental features described more recently in literature elucidating the stark variation in phenotypes evident in the *CACNA1A*-related HM population (19, 20). However, the extensive impact of *CACNA1A*-related HM events on patients and caregivers is understudied. Furthermore, HM events cannot be used as endpoints for clinical trials due to their paroxysmal nature. The experience from patients and caregivers can aid in identifying surrogate endpoints and parameters for future clinical trials to develop effective therapeutics, including gene therapy alternatives. Disease Concept Models (DCMs) are formal frameworks using qualitative interviews to depict the lived experience of a specific disorder, which can reveal novel phenotypes that impact treatment outcomes (21). A DCM covers three domains, including (1) symptoms, (2) symptom impacts on affected individuals, and (3) symptom impacts on caregivers. Unlike surveys that employ close-ended predefined queries around known features, this methodology allows open-ended questions to capture all aspects of the condition. DCMs have been established in other rare diseases, such as Dravet Syndrome, Angelman syndrome, and *STXBP1*-related disorders, which have resulted in an improved understanding of these disorders and the impacts of the comprehensive symptomology on both the patient and caregiver (21–23). Additionally, regulatory bodies, such as the FDA and the European Medicines Agency (EMA), have cited DCMs when determining the most appropriate surrogate endpoints for clinical trials (24, 25).

Although DCM development is underway for *CACNA1A*-related neurodevelopmental disorders as a whole, individuals with a *CACNA1A*-related HM diagnosis will only represent a small percentage of the individuals in this cohort. However, this subpopulation is distinct and often has a more severe and variable phenotype. Additionally, the paroxysmal nature of the HM episodes introduces potentially unique impacts to both the affected individual and the caregiver. Recent data investigating the longitudinal trajectory of HM events in a cohort of 15 individuals with *CACNA1A*-related HM reinforces the unpredictability of the episodes, which can be life-threatening (19). Therefore, this DCM is focused on *CACNA1A*-related HM using semi-structured qualitative interviews with male and female caregivers of children and adults with *CACNA1A*-related HM, uncovering 27 concepts within the domains of symptoms, symptom impacts, and caregiver impacts.

## 2 Materials and methods

### 2.1 Study design

As a DCM focused on *CACNA1A*-related HM, the research question concentrated on the impacts of HM events. This study used an inductive thematic approach to explore HM episodes and determine the impacts on the patients and caregivers during events and in their daily lives. Based on a previously reported systematic literature review of *CACNA1A-*related HM and a subsequent scoping review of caring for episodic and critically ill offspring, a semi-structured interview guide (Supplementary Document 1) consisting of 7 open-ended questions and two requested recommendations was created to enable honest dialogue with caregivers (20, 26–28). Experts in DCM methodology and *CACNA1A*-related disorders reviewed the questionnaire and provided feedback to confirm adequate content and clinical relevance.

### 2.2 Recruitment

Caregivers of patients with a confirmed diagnosis of *CACNA1A*-related HM were recruited through the *CACNA1A* Foundation, a patient advocacy organization, and their HM/Developmental Epileptic Encephalopathy (DEE) Support Group through the dissemination of a flyer describing the study on the Foundation’s website. Eligible caregivers were asked to engage in a 1-h interview through a virtual video platform. In order to remove bias due to potential miscommunication, the interviews were conducted in the native language of each participant. Caregivers of patient populations of pediatrics (1–11 years of age), adolescents (12–17 years of age), and adults (18+ years of age) were enrolled to capture the presentation of HM events over time and the subsequential impacts. Additionally, various sexes, marital statuses, countries of origin, and cultural backgrounds of caregivers were sampled to ensure a broad range in demographic status, healthcare diversity, and family dynamics. The research was performed in accordance with the Declaration of Helsinki. Ethical approval was provided by Clemson University’s Institutional Review Board (IRB2023-0802). Each caregiver verbally consented prior to the beginning of the questionnaire.

### 2.3 Data collection

Interviews were executed between March 11, 2024, and April 30, 2024, utilizing an open-ended semi-structured interview guide over a virtual platform to glean spontaneous narratives of experiences around HM episodes. The questions were not directing, and no specific symptoms were named except for HM to reduce potential interviewer bias and to illicit maximum participant-relevant information. More focused questions in the interview guide were asked to ensure the totality of concepts around a specific topic was garnered. The interviews were audio, video, and transcript recorded. The transcripts were de-identified and checked by the interviewer for accuracy of transcription. The authors entered the transcripts into DeDoose (Version 9.0.107), a HIPAA-compliant computer software that facilitates the itemization of qualitative data into groups.

### 2.4 Analysis

The interview transcripts were analyzed using DeDoose. An inductive thematic analysis approach was employed to ensure that all concepts identified were data-driven and not defined *a priori*, capturing topics crucial to caregivers (29–31). A coding tree was established prior to the analysis of transcripts to organize how concepts and sub-concepts would be distributed to the three domains of symptoms, symptom impacts, and caregiver impacts (Supplementary Document 2). Two researchers from the study team coded all of the interviews using a constant comparative method to ensure dependability (31).

Following coding all the transcripts, we calculated the frequency of certain concepts referenced in all interviews and those highlighted in most interactions. To detect differences in impacts based on the age of the patient, we stratified the prevalence of concepts based on defined lifespans, including childhood (1–11 years), adolescence (12–17 years), and adulthood (18+ years). Due to imbalances in the patient populations, the normalization function was employed through DeDoose software to equalize each stratum.

Conceptual saturation occurs during qualitative data collection and examination when novel information no longer substantially adds to the existing data (32–35). The sample size must adequately represent the target population and demonstrate confidence that the inquiry was fully answered to reach conceptual saturation. This study calculated concept saturation using the Guest et al. (32) methodology. A saturation table (Supplementary Document 3) was created employing a base size of 4, a run length of 2, and a new information threshold of <5% (30). Concept saturation was attained at 10 interviews, proposing a sufficient sample size with 13 interviews.

## 3 Results

### 3.1 Demographics

We transcribed 11 h and 9 min of discussion with 13 caregivers, yielding 2,018 references within 27 concepts (Tables 1, 2). Thirteen caregivers from two countries, the United States and Canada, were interviewed, including 11 females and 2 males. The marital status of the caregivers varied to capture multiple family living situations. These caregivers cared for a total of 12 individuals diagnosed with *CACNA1A*-related HM. Over half of the patients were non-speaking, and their ages ranged from childhood through adulthood (7–35 years old) (Table 3).

**Table 1:** Domain 1- symptoms and domain 2-symptom impacts: quotes from 13 interviews with caregivers.

Domain	Concept	Sub concept (if further delineated)	Quote
Symptom	Seizure	*“[our child] has not had a hemiplegic migraine outside of a status seizure. So, the status seizure comes first.”*	
	Apneic episodes	*“…because she stops breathing. And, because we have this experience, we are able to give her emergency medication, bag her, and get her to a steady state.”*	
	Subtle seizures	*“His seizures can be really subtle… the one time we used the EMS, he was having really subtle signs of seizing. He was turning blue. He was… really going downhill fast, …. And I was like, he is having a seizure…and they were like, oh, I do not think he is having a seizure.”*	
	Lost skills	-*“…Is she going to use a wheelchair for the rest of her life? Is she ever going to be able to talk again? … at that point… she could not even swallow, so it was constant drool and feeding tubes… is this what the rest of her days are going to be like?”*	
Symptom impact	Medical interventions	Urgency of HM Treatment	*“Her first major hemiplegic migraine was unrecognized… not treated quickly, and left brain damage…her not able to walk… lost all her words and changed our life.”*
	Health impact	Regression	*“He has declined, he has lost skills, he has lost abilities. … right now, he does not have a quality of life.”*
	Emotional impact	Anxiety/Fear	*“He said do not call, do not call, because he did not want me to call 911 because he does not… because it is always just so terrible.”*
		Anger/Sadness	*“When she is… in the thick of it, she is just so miserable …She does not like being hooked up to the EEG… So, we typically do not do EEGs … except, at the beginning of January …and she balled for 3 h.”*

**Table 2:** Domain 3- caregiver impacts: quotes from 13 interviews with caregivers.

Concept	Sub concept	Quote
Caregiver requirements	Advocacy	*“Being a good advocate is important and not backing down.”*
	Care at Home	*“Once we got better control … rescue meds working… we administered everything … called 911… if it stopped, we would not take him to the hospital … we had been to the hospital so many times… and there is a price that everybody pays.”*
Emotional impact	Worry	*“There is a lot of fear… when might the next one come. Will we be able to get the treatments… in the time… to protect her brain from the permanent damage?”*
	Burden	*“This neurological event. … it is on your shoulders…the burden is on you to figure out what is going on and how do I identify it and how to get physicians to listen to you about what is going on…”*
	Grief	*“There is a true grief you feel because there are losses. He is still here. But you have lost part of him.”*
HM treatments	Listening to caregiver	*“I was taking her to the neurologist… I think they are hemiplegic migraines… she says, no, they are not… I said but she had that diagnosis. [The doctor states]“She needs to see a psychiatrist…This is all psychological.” I said, no, it is not.”*
	Transportation	*“We have used [EMT/Ambulance] once…we used it for her first status episode. They wound up getting lost…my son had to go chase them…So now we just load and go.”*
		*“The couple of times we have had to use it. They have been terrific. Because that is what they are trained to do.”*
	Triggers	*“When we got the hemiplegic migraine diagnosis… I was able to go back in time…some of the worst-case scenarios where they said he was concussed…it was the paralysis that caused the concussion.”*
Daily living impact	Formal Support	*“[Parents] have to make sure they have an emergency plan. It has got to be written… that piece of paper has changed things.”*
		*“And so, what I have started to do is since the CACNA1A Foundation has been created, I go to their… medical provider tip sheet. And I always tell them go to this website, read it, here is everything you need to know.”*
	Disruption	*“We wanted to live within a radius of the hospital because when we lived in…, it was an absolute nightmare to get to a hospital.”*
Health support	HCP Knowledge	*“So, his neurologist…within our first couple months of him being seen at [X], we had a diagnosis.”*
Access to care	Information/Education	*“I do not get…security called on me anymore. I know how to handle myself so that I will be heard”*
Social impacts	Sibling/Family	*“She reminds him [brother] of flowers because they are so delicate and fragile…they can survive rainstorms… that [was] so be beautiful… oh my God, you know.”*
	Socialization	*“We rarely travel with her. When we do it is care very carefully planned.”*
Resilience	Coping	*“Live even though it is not normal…just to keep things like as normal as possible…You just have to get to a point in your life where… this is what we do. This is why we do it… it is just your normal”*
		*“The counselor was really good…you do not want to poke the bear and wake all that stuff up… It is kind of like seizure control. We do not mess with any meds if things are working… we do not poke the bear…”*
Discrimination	Racism	*“As you know, a black family, we hear a lot in the media about like, how African-Americans are treated in the hospital…I instantly go into those settings feeling like I have to really advocate for my child. Much stronger because we are a black family.”*
	Ableism	*“There is definitely, I want to say, prejudice for lack of a better word… Our kids the frequent flyer… Our kid maybe is not …going to rule the world… that does not mean we do not want the best for her. So, overcoming those prejudices.”*

**Table 3:** Demographics of caregivers and individuals with *CACNA1A*-related HM.

Caregivers (*n* = 13)		Individuals with *CACNA1A*-related HM (*n* = 12)	
Sex	*n* (%)	Sex	*n* (%)
Male	2 (15.4%)	Male	7 (58.3%)
Female	11 (84.6%)	Female	5 (41.7%)
Lineage		Lineage	
White	12 (92.3%)	White	11 (91.7%)
Non-white	1 (7.7%)	Non-white	1 (8.3%)
Marital status		Age at interview	
Married	11 (84.6%)	Childhood (1–11 years)	5 (41.7%)
Divorced	2 (15.4%)	Adolescence (12–17 years)	5 (41.7%)
Country		Adulthood (18+ years)	2 (16.6%)
USA	11 (84.6%)	Country	
Canada	2 (15.4%)	USA	10 (83.3%)
		Canada	2 (16.7%)
		Communication	
		Verbal/Some words	5 (41.7%)
		Non-speaking	7 (58.3%)

### 3.2 Symptoms

**Figure 1:**
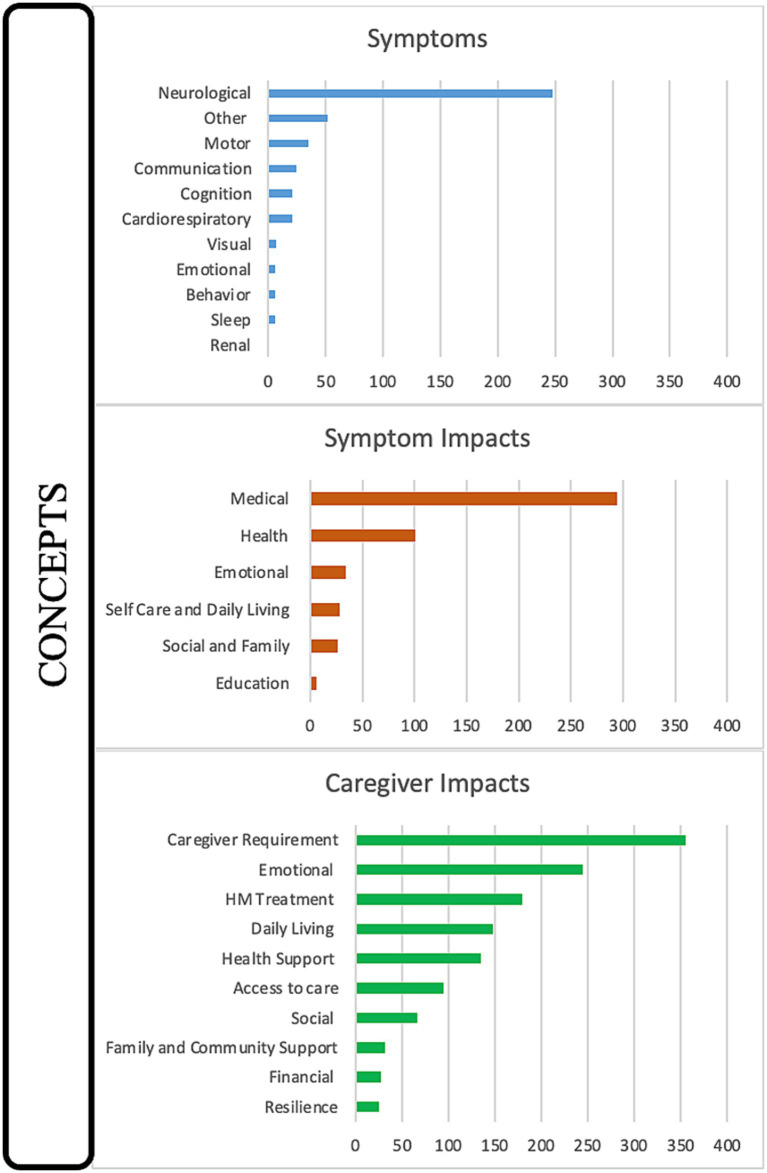
Disease concept model for *CACNA1A*-related HM. Twenty-seven distinct concepts were identified from 2,018 references during 13 interviews with caregivers and categorized into the domains of Symptoms, Symptom Impacts, and Caregiver Impacts.

**Figure 2:**
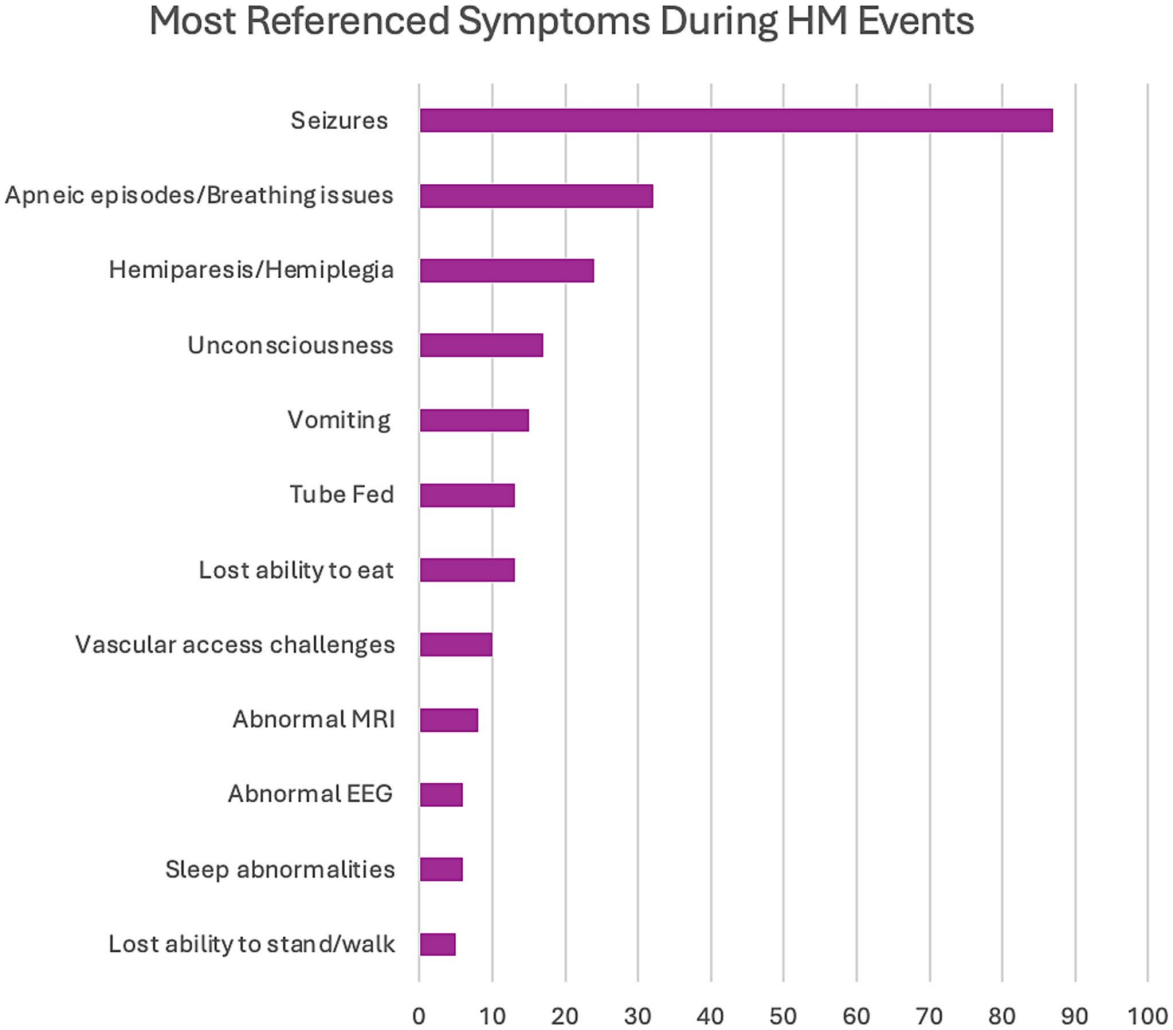
Most referenced symptoms during HM events.

The first domain consisted of the identification of symptoms reported by caregivers primarily during HM events but also included some baseline characteristics. The complete range of referenced symptoms covered 11 discrete concepts (Figure 1). The severity of symptoms (58 references across 92.3% of interviews) and variation in presentation (45 references across 76.9% of interviews) were mentioned in most interviews, demonstrating the range of manifestations that can be seen in the same patient throughout a lifetime. Seizures were the most referenced symptom, at 87 times across almost all interviews (84.6%) in conjunction with HM episodes either preceding or post (Figure 2). The most common seizure-related manifestation seen proximal to HM was status epilepticus, mentioned 27 times. Caregivers reported other well-recognized symptoms during HM episodes, including hemiparesis/hemiplegia (24 references across 76.9% of interviews), unconsciousness (17 references across 38.5% of interviews), and vomiting (15 references across 30.8% of interviews).

We found a serious symptom during HM events, apneic episodes or breathing difficulties (32 references across 61.5% of interviews), that may have been underreported in the literature. Apnea during HM events was previously reported as present in 4 of 33 cases (12.1%) in our earlier systematic literature review of *CACNA1A*-related HM.22 Also, multiple caregivers (*n* = 4) cited seizures that were difficult to detect by emergency technicians and emergency room medical personnel (referenced six times), which is something that has not been documented in the literature with *CACNA1A*-related disorders. Some other significant symptoms that have not been highlighted in the literature include lost ability to eat (13 references across 53.8% of interviews), tube feeding (13 references across 38.5% of interviews), vascular access challenges (10 references across 53.8% of interviews), and lost ability to stand/walk (5 references across 23.1% of interviews). Although many of these skills can be re-learned, it may require months or rehabilitative therapies. Of note, two patients have permanent deficits post HM events regarding ambulation; one patient lost the ability to walk, and the other can no longer stand. Regarding vascular access, three patients required port placements, and two individuals needed intraosseous (IO) access.

### 3.3 Symptom impacts

The symptoms of HM affected the daily lives of the individuals suffering from the disease. The totality of symptom impacts (domain two) spans six concepts, including medical (294 references), health (101 references), emotional (34 references), self-care/daily living (28 references), social/family (26 references), and education (6 references) (Figure 1). Medical impacts referred to actions the patients had to take to treat the HM events while health impacts referred to the bodily effects of the HM events. The most prominent symptom impact was medical, with all interviews referencing HM treatment (140 references), often coded with or co-coded with seizure treatment (53 references), the urgency of appropriate HM treatment (49 references), and rescue medication (40 references). Surgical/medical interventions in the hospital (60 references across 69.2% of interviews) were also coded with or double-coded with HM treatment and frequently co-coded with intubation (26 references). Two other common double codes were side effects (30 references) and medication failure (22 references).

Within HM events, triggers are often discussed (27 references across 76.9% of interviews) due to a definitive cause yet to be identified. The most common triggers co-coded with HM events were seizure (7 references), dehydration (4 references), and stress (4 references). Of note, head trauma, which has been a documented trigger in the literature, was mentioned by two caregivers, questioning whether it was the cause of the HM event or a result of the hemiparesis/hemiplegia that occurred first and caused the affected individual to fall.

HM events also impacted health substantially and were referenced by all caregivers, some temporarily (*n* = 9 individuals) and others with permanent regression (*n* = 3 individuals). Recovery from HM events is often slow (16 references across 58.3% of interviews), frequently reported to be due to the severity of illness (13 references across 58.3% of interviews). The deterioration in health, whether transient or enduring, resulted in impacts on self-care and daily living, including regression and re-learning activities of daily living (ADLs) (19 references across 58.3% of interviews).

Of note, seven affected individuals were non-speaking, and most of the remaining speaking individuals lacked the ability to express their feelings (12 references across 30.8% of interviews, affecting 60.0% of verbal individuals). Therefore, the emotional impacts on affected individuals were far less captured across the interviews, creating a significant knowledge gap. Anxiety was the most referenced emotional impact (13 times, across 58.3% of interviews). Also impactful to the affected individuals were fear (9 references across 38.5% of interviews) and frustration (7 references across 15.4% of interviews). Pain post-HM was also cited eight times by 30.8% of caregivers. Anger (4 references across 23.1% of interviews), sadness (3 references across 23.1% of interviews), and desire to be released from the hospital (3 references across 15.4% of interviews) were also apparent in affected individuals after HM events.

### 3.4 Caregiver impacts

Domain three, caregiver impacts, was featured most prominently in the interviews, mentioned in 100% of interviews and accounting for approximately 50% of the discussion time, including 995 references and 10 distinct concepts. All caregivers described the concepts of caregiver requirements (355 references), emotional (245 references), HM treatments (179 references), daily living (148 references), health support (135 references), access to care (95 references), and social (67 references). Slightly less impactful, referenced in half of all interviews, were family/community (32 references), financial (28 references), and resilience (25 references) (Figure 1).

#### 3.4.1 Caregiver requirements

Caregiver requirements were referenced 355 times across all interviews and were often coded with advocacy role (174 references), knowledge of how to care for *CACNA1A*-related HM (59 references) and fight for appropriate, timely treatment (34 references). Also, co-coded with caregiver requirements was the medical care provided by the caregiver at home (45 references) and was also linked with the goal of avoiding the ER and hospitalization (17 references).

#### 3.4.2 Emotional impacts

Emotional impacts were referenced 245 times by all caregivers and were co-coded most often with anxiety and stress (95 references), followed by fear of the medical unknown (40 references). Anxiety and stress were double coded with “worry,” including worry about (a) the next HM episode (40 references across 92.3% of interviews), (b) an HM episode being treated incorrectly (15 references across 53.8% of interviews), and (c) disease progression/regression (14 references across 46.2% of interviews). Another emotional effect of HM events on caregivers is “burden”: the burden to recognize an event early (19 references, 53.8% of interviews) and the burden to keep their child safe (13 references, 46.2% of interviews). Although not referenced as frequently as some of the other emotional impacts, grief was noted (10 references).

#### 3.4.3 HM treatments

The experiences in the emergency rooms and hospitals were reflected by referencing HM treatments 179 times across all interviews. Medical providers were double coded with HM treatments and were referenced 148 times across all interviews. Medical providers were also frequently co-coded with a lack of information on *CACNA1A* (114 references, by 100% of interviews), misdiagnosis of HM event (28 references across 76.9% of interviews), listened to the caregiver (23 references across 84.6% of interviews), and did not listen to the caregiver (13 references across 46.2% of interviews). Also, double-coded with HM treatments was transportation to the hospital, including both negative (13 references across 46.2% of interviews) and positive experiences (23 references across 53.8% of interviews). Discrimination (3 references across # interviews) was discussed within HM treatments as it related to care. It addressed both racism and ableism.

#### 3.4.4 Daily living impacts

Daily living impacts were referenced 148 times across all interviews and were co-coded most frequently with a need for a formal support system (70 references), specifically an emergency protocol (57 references). The other common double code for daily living was disruption, referenced 32 times across 76.9% of interviews.

#### 3.4.5 Health support and access to care

Health support was cited 135 times by all caregivers and was frequently double-coded with shared decision-making (33 references) and Healthcare Practitioner (HCP) with knowledge of the disorder (23 references). Access to care was referenced 95 times by all caregivers and was most frequently co-coded with Information/Knowledge (49 references). The caregivers discussed the learning curve needed to navigate the system. Also double-coded was the healthcare provider (consistent contact) (25 references), who assisted in educating and supporting the caregivers.

#### 3.4.6 Social impacts and resilience

Referenced 67 times across all interviews, social impacts focused on the effects on siblings and family (45 references) and reduced socialization/leisure activities (19 references). Under resilience, although referenced less frequently, coping strategies were mentioned 20 times across 61.5% of all interviews and were most often co-coded with “restoring your normal” (5 references across 38.5% of interviews) and compartmentalization (5 references across 23.1% of all interviews), which allows a “survival mode” mentality to focus on the needs of the patient.

### 3.5 Caregiver responsibilities differ by gender

Mothers and fathers were attuned to the needs of the child, but the roles differed. Based on descriptions of HM events, mothers appear to function more often as the primary caregivers (12 references) and take on the role of learning how to care for *CACNA1A*-related HM and understanding disease management. Fathers’ roles were supportive, including maintaining household finances, providing emotional encouragement, helping with advocacy, and running to and from home for supplies.

### 3.6 Most referenced *CACNA1A*-related HM concepts stratified by age group

**Figure 3:**
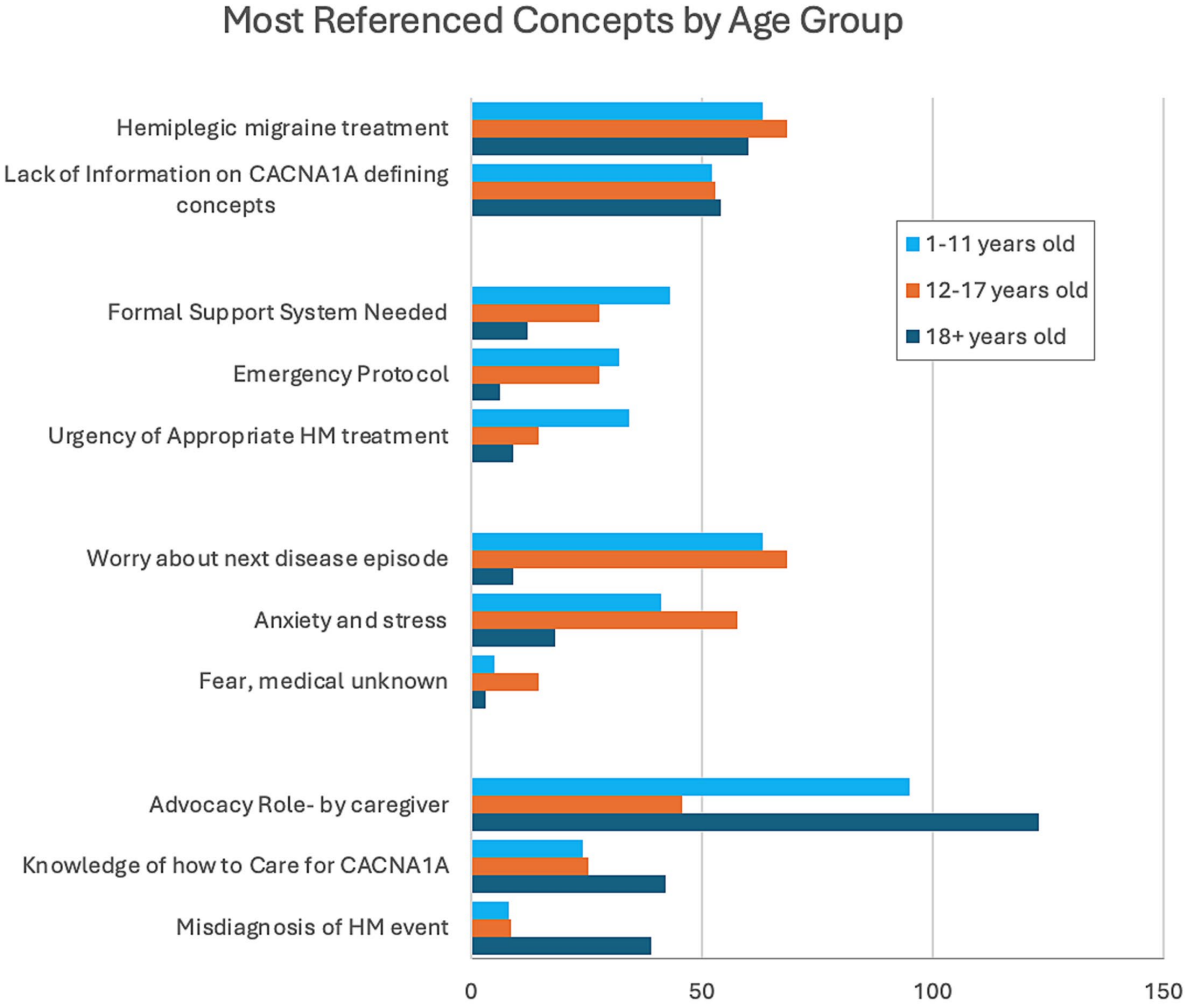
Distribution of concepts stratified by age.

To evaluate if the impacts of *CACNA1A*-related HM events change over time, we stratified the most referenced concepts by age groups of the individuals: childhood (1–11 years), adolescence (12–17 years), and adulthood (18+ years) (Figure 3). HM treatments and lack of information on *CACNA1A* were referenced by all age groups equally. Caregivers of individuals in childhood referenced the urgency of appropriate HM treatments, the need for a formal support system, and emergency protocols more frequently. Anxiety and stress, specifically worry over the next disease episode, were prevalent in discussions with parents of adolescents. For caregivers of adults, knowledge of how to care for *CACNA1A* and the misdiagnosis of HM events were overrepresented references. Also stressed in this group was advocacy due to the caregivers’ belief that transitioning from pediatric to adult medicine was like resetting care to start anew.

## 4 Discussion

We employed DCM methodology to establish the oscillating topography of HM events in symptoms and experiences, revealing the impacts of *CACNA1A*-related HM episodes and emergency measures on patients and caregivers (21–23, 36). In addition, these concepts uncovered challenges faced by caregivers in handling HM events and yielded key learnings for better management techniques in the future. The discussion of HM events centered on the severity of symptoms and variation in presentation from episode to episode, demonstrating the diversity of presentation based on the gravity of the event. This reinforces what was found in our recent longitudinal investigation of 15 patients with *CACNA1A*-related HM, demonstrating the unpredictable and often severe nature of the disorder (19). The referenced symptoms focused primarily on HM events. They covered 11 concepts, including features not fully investigated or described in the literature, such as severe regression and vascular access challenges.

### 4.1 Findings

This study has four main findings and implications for further research. To begin, published clinical characterizations of *CACNA1A*-related HM have only captured a small subset of the lived experiences of patients, caregivers, and family members. Although recent clinical descriptions of *CACNA1A-*related HM have substantially broadened the phenotype, the full impact of HM episodes, the salient feature of this disorder, on individuals and their families has not been evaluated thus far. Our data shows that the impacts of these events extensively affect the individual and caregiver daily, as was demonstrated by 59.3% of all concepts identified as being associated with these two domains. Future studies are needed to better measure outcomes from these two domains, specifically quality of life. Several studies have validated measurement models used to obtain the impacts of other diseases on daily living, and work is ongoing regarding capturing quality of life in patients with developmental and epileptic encephalopathies (37–39).

### 4.2 Symptom impacts

Secondly, the impacts of HM events on the individual and caregiver are often devastating. Symptoms result in many patients needing to re-learn basic skills that they possessed prior to an HM event. Unfortunately, if not treated early and appropriately, these events can leave a patient permanently altered with cognitive and physical regression (3/12, 25%), impacting the quality of life for both the patient and caregiver, as well as the entire family. These events need to be treated early with appropriate therapy to offer individuals the best hope of making these impacts transient in nature. In addition, emotional impacts on patients are underreported due to the overall communication challenges of the population, with many being non-speaking or otherwise unable to express what they are feeling. Better tools are needed to assess the emotional impact of these events on patients. Caregivers demonstrated extensive knowledge of their child’s condition and made concerted efforts to become educated in order to advocate for their child. Additionally, the data highlights the sacrifices made by the caregivers in their choices of what they do and where they live in order to maximize optimal treatment.

### 4.3 Impacts stratified by age

The third finding highlights the variation in caregiver impacts based on the age group of the affected individual. Caregiver impacts are vast, and although we captured many of the issues they face daily, this study only superficially discusses what can and should be investigated in the future. The only uniformly referenced concepts by caregivers of all ages are the consistent need for HM treatment and the obscurity of *CACNA1A*-related HM among healthcare providers. Our findings show that caregivers of children (1–11 years) are faced with a new disorder that is unfamiliar to them and to the general medical community. They are initially unequipped but are driven to learn about the disease quickly and then advocate for early appropriate treatment for their child. Caregivers referenced their best tool as a written emergency protocol, which they always carry to guide ER doctors and staff who are unfamiliar with *CACNA1A*-related HM. Sometimes, the protocol can appear aggressive, and often, the burden of explaining the severity of symptoms and the urgency needed to treat still falls on the caregiver. Caregivers of adolescents (12–17 years) spoke about anxiety and stress, worrying about the next event due to the evolution of the disorder as the patient ages. They discussed changes in how the HM events present, resulting in fear of the medical unknown. The transition from pediatric care to adult (18+ years) care posed many problems for caregivers of adult patients. Care was transferred from clinicians knowledgeable about the disorder and hospitals who knew their children and how to care for them during HM events to clinicians unfamiliar with *CACNA1A*-related HM. They again referenced the advocacy role, but this time, the caregiver knows how to care for *CACNA1A* and wants to share that knowledge with clinicians. This group referenced the misdiagnosis of HM events more frequently and the resistance to acknowledge them.

### 4.4 Transition from pediatrics to adult medicine

Our data suggest that the passage to adulthood means losing knowledgeable clinicians and hospitals, replaced by healthcare providers and clinics new to their rare disease. These new providers need to be trained to best care for these individuals. Pediatric neurologists and other specialties must educate adult practitioners on managing these disorders. Other studies have investigated the issues faced by individuals with rare diseases transitioning care and have identified multiple challenges that require further examination for better resolution (40, 41). Although some suggestions have been made for improved transition to adult medicine, caregivers need to be better educated on navigating the healthcare system as well as improved identification of adult practitioners knowledgeable in *CACNA1A*-related disorders.

### 4.5 Practice implications

Given the rarity of the disorder and the challenges with the transition from pediatrics, there are two important opportunities for improved management of HM. Findings suggest appropriate and timely treatment to minimize permanent adverse outcomes such as enduring cognitive and physical regression (16, 42). Firstly, a transition plan for adolescents approaching adulthood is warranted. Providers can benefit from collaboration with pediatric neurologists who are familiar with the case. Secondly, adding a written emergency protocol from the patient’s neurologist can guide treatment in other healthcare settings. Clinicians may not be well-versed in the management of *CACNA1A*-related HM. The protocol can empower caregivers with a document to guide emergency medical care for individuals with a *CACNA1A*-identified variant. Due to the unpredictability and severity of the disorder, an identified variant is enough to carry out an emergency protocol. 76.9% of caregivers reported positive responses to emergency protocols signed by neurologists, removing the burden of advocating for appropriate, timely treatments. Written protocols may initiate shared decision-making and create a partnership in care for their loved one. Medical professionals may be more receptive to guidelines proposed by other clinicians who are more experienced in managing a disorder.

### 4.6 Limitations

Recruitment for this study came exclusively from the CACNA1A Foundation, although a global organization, this fact may have skewed the severity of the patient population involved in the study. Often more mild cases are less likely to seek out the support of an advocacy group. With that in mind, many of the individuals with *CACNA1A*-related HM in this investigation were non-speaking or lacking the ability to report on their experiences and therefore, the findings of this study came solely from caregivers and not individuals themselves. Another ramification of this limitation may be underreported symptom impacts. Although open-ended questions in semi-structured interviews are not free of bias, we strived to reduce prejudice by capturing spontaneous narratives using non-directing probes. However, since the responses were from caregivers and not the patients directly, caregiver bias was possible. As stated earlier, *CACNA1A*-related HM is a heterogenous phenotype and in mild cases, often seen with FHM1, patients can be neurotypical with the ability to self-report. Future studies should include this population for a more comprehensive look at impacts of HM events to patients. Also of note, all individuals and their caregivers live in North America, which may not reflect the experiences of *CACNA1A*-related HM patients globally or the experiences of other healthcare systems. Finally, due to the small sample size, some concepts may be referenced more in different groups of individuals, and patient experiences may vary.

### 4.7 Conclusion

This novel study established a DCM for *CACNA1A*-related HM, revealing 27 distinct concepts (Figure 1) representing the lived experiences of families with *CACNA1A*-related HM. We captured the variable landscape of HM events relative to symptoms and experiences, uncovering the impacts of *CACNA1A-*related HM episodes and emergency treatments on patients and caregivers. Additionally, we identified previously underreported and unreported symptoms related to HM events, knowledge gaps in symptom impacts based on communication challenges within the *CACNA1A*-related HM population, and more impactful concepts for caregivers based on the patient’s age. Two opportunities to improve patient management were also identified, including a transition plan for adolescents approaching adulthood and a written emergency protocol. The establishment of a disease model for *CACNA1A*-related HM, for which there are no specifically designed FDA-approved treatments, is helpful in identifying surrogate endpoints for future clinical trials by highlighting aspects of the disease that have the most impact to patients and caregivers.

## Data Availability

The original contributions presented in the study are included in the article/Supplementary material, further inquiries can be directed to the corresponding author.
