# Supplementary material for: “Living with” CACNA1A-related hemiplegic migraine, a disease concept model
Source: Front Neurol. 2024 Nov 1;15:1460187. doi: 10.3389/fneur.2024.1460187 (PMC11565606; doi:10.3389/fneur.2024.1460187)
Supplement: Supplementary file 3 [file Data_Sheet_1.PDF]

Interview Guide

1. How has your life changed since experiencing your loved-ones first hemiplegic migraine?
2. How do you decide when you need to go to the Emergency Room (ER)?
3. What is an ER visit like?
    a. What, if anything, do you bring?
    b. If you use EMT/Ambulance, what is that like?
    c. What happens when you arrive?
        i. Home ER?
        ii. Away ER?
4. How often does the ER visit become an ICU admission? What is that like?
5. What is the Intensive Care Unit (ICU) admission and stay like?
    a. Tell me about a day there?
    b. Emotions and Emotional Support?
6. How do things change when you return home?
7. How does everyone returning home impact each of the family members?

Recommendations:

A. What do you wish the ER staff knew to make this process easier for your loved one? For you?

B. What do you wish the ICU staff knew to make this process easier for your loved one? For you?

Instructions for the interviewer:
The following interview guide is designed as a semi-structured format where the interviewer will not ask leading
questions but instead explore points that the participant raises through non-leading probes. Some examples of
non-leading probes include:

- Can you describe exactly how that feels?
- Tell me more about that.
- How does that affect you?
- Can you talk more about ___________?
- How often does that happen?
- How long does that last?
- Is there anything that makes it better or worse?
- How do you cope with that?
- What makes you say that?

It may not be necessary to ask every question in this guide (i.e., if the participant does not list seizures as a part of
their clinical picture, then we do not need to ask questions related to seizure history).
Questions in this guide may be explored in any order.
